# Temperature-Arousing Self-Powered Fire Warning E-Textile Based on ***p***–***n*** Segment Coaxial Aerogel Fibers for Active Fire Protection in Firefighting Clothing

**DOI:** 10.1007/s40820-023-01200-8

**Published:** 2023-10-13

**Authors:** Hualing He, Yi Qin, Zhenyu Zhu, Qing Jiang, Shengnan Ouyang, Yuhang Wan, Xueru Qu, Jie Xu, Zhicai Yu

**Affiliations:** 1 State Key Laboratory of New Textile Materials and Advanced Processing Technologies, School of Textile Science and Engineering, Wuhan Textile University, Wuhan, 430200 People’s Republic of China; 2 National Local Joint Laboratory for Advanced Textile Processing and Clean Production, Hubei Key Laboratory of Biomass Fibers and Eco-Dyeing and Finishing, Wuhan Textile University, Wuhan, 430200 People’s Republic of China; 3 Jiangsu New Horizon Advanced Functional Fiber Innovation Center Co., Ltd., Suzhou, 215000 People’s Republic of China

**Keywords:** Self-powered fire warning, Coaxial wet spinning, *P*–*n* segment thermoelectric fiber, Thermoelectric textiles, Active fire protection

## Abstract

Firefighting protective clothing is a crucial protective equipment for firefighters to minimize skin burn and ensure safety firefighting operation and rescue mission. A recent increasing concern is to develop self-powered fire warning materials that can be incorporated into the firefighting clothing to achieve active fire protection for firefighters before the protective clothing catches fire on fireground. However, it is still a challenge to facilely design and manufacture thermoelectric (TE) textile (TET)-based fire warning electronics with dynamic surface conformability and breathability. Here, we develop an alternate coaxial wet-spinning strategy to continuously produce alternating *p*/*n*-type TE aerogel fibers involving *n*-type Ti_3_C_2_T_*x*_ MXene and *p*-type MXene/SWCNT-COOH as core materials, and tough aramid nanofiber as protective shell, which simultaneously ensure the flexibility and high-efficiency TE power generation. With such alternating *p*/*n*-type TE fibers, TET-based self-powered fire warning sensors with high mechanical stability and wearability are successfully fabricated through stitching the alternating *p*–*n* segment TE fibers into aramid fabric. The results indicate that TET-based fire warning electronics containing 50 *p*–*n* pairs produce the open-circuit voltage of 7.5 mV with a power density of 119.79 nW cm^−2^ at a temperature difference of 300 °C. The output voltage signal is then calculated as corresponding surface temperature of firefighting clothing based on a linear relationship between TE voltage and temperature. The fire alarm response time and flame-retardant properties are further displayed. Such self-powered fire warning electronics are true textiles that offer breathability and compatibility with body movement, demonstrating their potential application in firefighting clothing.

**Supplementary Information:**

The online version contains supplementary material available at 10.1007/s40820-023-01200-8.

## Highlights

- The *p*–*n* segment thermoelectric aerogel fiber was fabricated through an alternating coaxial wet-spinning strategy.
- Resultant alternating *p*–*n* segment thermoelectric fiber was electrically connected in series (two *p*–*n* pairs with a length of 3 cm) with an outstanding electrical conductivity of 23.76 S m^−1^.
- Thermoelectric textile-based self-powered fire warning electronics exhibited sensitivity (trigger time within 1.43 s) and repeatable temperature sensing performance for firefighting clothing.

**Supplementary Information:**

The online version contains supplementary material available at 10.1007/s40820-023-01200-8.

## Graphical abstract

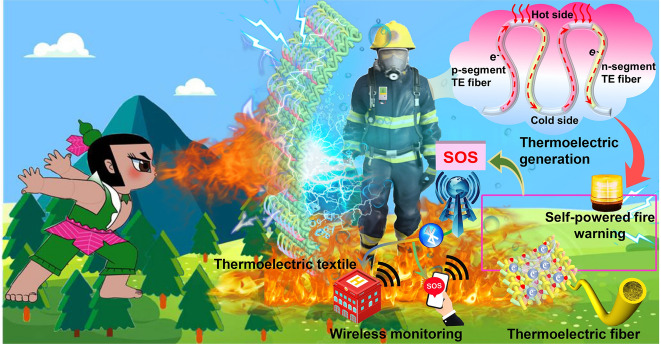

## Introduction

Fire is an important form of energy that sustains the global ecosystem and is also the source of human civilization, which bringing human beings into the age of civilization. Over millions of years of human evolution, humans have feared fire, worshiped it, used it, and loved it. It is an indisputable fact that the advancement of human society is accompanied by the control and utilization of fire [1–3]. The uncontrolled fires can cause life casualties, enormous property losses and serious environmental pollution to global ecosystem. Therefore, fire safety issues have raised considerable concerns in modern society, particularly with the ubiquitous application of flammable materials in building construction or home decoration [4]. Firefighters are one of special high-risk occupational groups that struggle with fire and protect the lives and property of the public. In China, firefighters are called the “highest esteem people” and are always the first to quickly respond to fire accidents. The latest statistics from the National Fire and Rescue Service revealed that around 825,000 fire accidents took place throughout China in 2022 and resulted in the deaths of 25 firefighters (including forest fires and high-rise building fires) [5–7]. Firefighting protective clothing serves as a crucial barrier between firefighters and fire hazards, playing a vital role in minimizing skin burns from high temperatures or flames and ensuring the safety of firefighters during firefighting operation and rescue mission [8, 9]. Unfortunately, aramid fiber, which is the main substrate of firefighting clothing, will also undergo thermal decomposition when the temperature exceeds 500 °C, resulting in the failure of the firefighting clothing to provide thermal protection to firefighters [10]. Therefore, it is urgent to develop effective fire safety strategies to safeguard firefighters from getting burned while conducting fire and rescue operations, particularly when exposed to extremely high temperature and flame.

Currently, the effective strategies for safeguarding firefighters on fireground are primarily categorized as active fire protection and passive fire protection [11]. Generally speaking, passive fire protection for firefighters mainly aims to improve the thermal protection performance of firefighting clothing by increasing the layers and thickness of protective garment. However, this strategy will result in an increase in the weight of the firefighting protective clothing and reduce its heat dissipation and moisture permeability capabilities. Consequently, the operational efficiency of firefighters in a fire situation will be significantly compromised [12, 13]. In comparison, active fire safety protective strategy for firefighter involves an early fire warning sensor that can be integrated into firefighting clothing and monitor the surface temperature of firefighting clothing on fireground (≥ 400 °C). The integrated fire alarm system can quickly detect high temperature flame and provide an early warning for firefighters before firefighting clothing exceeds its own thermal decomposition temperature [14–16]. Thus, firefighters can actively evacuate to a safe distance from the fire before the firefighting protective garment becomes damaged and ensure their safety. This active fire safety protective concept for firefighting clothing was first proposed by Yu and He and co-workers [17]. Such a new type of intelligent fire warning sensor has quickly attracted the interests of researchers to achieve fire safety protection for firefighters. At present, a number of researchers focus on the resistance change-based sensors, which are mainly based on graphene oxide (GO) and semiconductor materials [4]. The former is that the electrically insulated GO can exactly remove the oxygen-containing groups and form the highly conductive reduced GO once encountering an abnormal temperature. By detecting the resistance changes of GO-based fire warning material, it can realize one-off early fire detection before the ignition of combustible materials [18–20]. Another typical semiconductor-based fire warning material exhibits a similar but reversible resistance transition under high temperature or flame, resulting in a repeatable fire warning response before the fire accident of combustible materials occur or resurgence [21–24]. Unfortunately, the above-mentioned resistance-type fire warning sensors heavily depend on external batteries for power, which cannot meet the needs of long-term service [25–27]. Meanwhile, the use of tough batteries not only presents challenges in integrating them into firefighting clothing but also increases the complexity and instability of the fire alarm system, especially under a fire circumstance. Therefore, it is still a huge challenge to fabricate a wearable self-powered temperature sensor for fire alarming systems without external power supply in firefighting clothing.

Thermoelectric (TE) textile (TET) shows promise in addressing the aforementioned challenge toward to truly manufacture wearable self-powered fire warning sensors with the advantages of miniaturization and long steady-state operation periods [28, 29]. In comparison with thermistor-type fire warning materials, TE material-based fire warning sensors could directly convert heat into electricity signal once encountering fire due to carriers in TE materials migrate along temperature gradients. Accordingly, the current flow goes through the fire alarm system and triggers a rapid-fire alarm signal without external power supply [30–33]. However, the utmost challenge involves developing rational ways to facilely design and continuously produce alternating *p*–*n* segment TE fibers for weaving TET at a large scale and high TE property. Owning to their good wearability of TET, including dynamic surface conformability, breathability, washability and unobtrusiveness, flexible fiber-based TETs have become a research frontier for manufacturing next generation wearable self-powered fire warning electronics [34–37]. Till now, most alternating *p*–*n* segment TE fibers are realized by dip coating or spraying on regular fibers with alternating *p*–*n* segment layers for TE generator [38–40]. However, these processes inevitably result in instability of the TE layer and easy fray/wear out with prolonged mechanical friction and deformation caused by body movement [41, 42]. In contrast to the above-mentioned approaches, coaxial wet spinning is a feasible method of producing continuous alternating *p*–*n* segment TE fibers with a protective shell structure to balance the mechanical and TE performance [43–45]. This configuration can seamlessly manufacture periodically in-series interconnected *p*–*n* segments along fiber and optimize TE characteristics, making them a primary choice for designing wearable self-powered fire warning textile through weaving TE fibers into a fabric.

Herein, we reported a feasible approach to fabricate temperature-arousing self-powered fire warning sensors based on *p*–*n* segment coaxial aerogel fibers for active fire protection in firefighting clothing. An alternating wet-spinning strategy was adopted to continuously and largely fabricate *p*/*n*-type segment TE fibers with alternating *n*-type Ti_3_C_2_T_*x*_ MXene (power factor of 0.098 μW m^−1^ K^−2^) and *p*-type MXene/SWCNT-COOH (power factor of 0.179 μW m^−1^ K^−2^) as core materials, and tough aramid nanofiber as a protective shell to ensure the flexibility and high-efficiency TE power generation. In this configuration, the alternating *p*–*n* segment TE fibers are electrically connected in series (two *p*–*n* pairs with a length of 3 cm) with an outstanding electrical conductivity of 23.76 S m^−1^. Through stitching the alternating *p*–*n* segment TE fiber into a flexible aramid fabric, TE textile can perform early-stage fire warning function for firefighting clothing and shows an output voltage of 7.5 mV at Δ*T* = 300 °C when the TET is exposed to a fire. TET-based fire warning sensor displays an accurate and repeatable temperature detecting performance within a wide temperature range of 100–400 °C, which triggers the fire alarm system within 1.43 s. More importantly, the resultant self-powered fire warning device is compatible with firefighting clothing and conforms dynamic body movement due to its excellent flexibility and mechanical properties. This work is promising in delivering a self-powered fire warning e-textile for active fire protection of firefighting clothing on fireground.

## Experiments

### Materials

PPTA (poly(para-phenylene terephthalamide)) fibers were supplied by Shanghai Mingxi Industrial Co., Ltd. Ti_3_AlC_2_ powder (200 mesh, ≥ 98.0%), sericin, dopamine hydrochloride (DA, 98%) and lithium fluoride (LiF) were purchased from Shanghai Macklin Biochemical Co., Ltd. Tertiary butyl alcohol, sulfuric acid (H_2_SO_4_), ammonium chloride (NH_4_Cl), potassium hydroxide (KOH), nitric acid (HNO_3_), hydrochloric acid (HCl) and dimethyl sulfoxide (DMSO) were provided by Sino Pharm Chemical Reagent Co., Ltd. Montmorillonite (MMT), silver nanowires (Ag NWs, purity > 99.5%, diameter of 50 nm and average length of 100 μm) and single-walled carbon nanotube (SWCNT, purity > 50%, diameter of < 2 nm and average length of 5–30 μm) were acquired from Aladdin Co., Ltd.

### Fabrication of Aramid Nanofibers (ANFs)/MMT Spinning Dopes

In this study, a proton-donor-assisted deprotonation method was employed to dissolve aramid fibers (PPTA) [46, 47]. Specifically, KOH (1.5 g) and PPTA (1.5 g) were added into a beaker with distilled water (3 mL) and DMSO (80 mL), followed by magnetic stirring for a week. After that, a viscous dark red ANFs suspension with a concentration of 18 mg mL^−1^ was obtained. Subsequently, 0.2 g of MMT power was slowly added into 1 mL of DMSO. The MMT/DMSO suspension was mechanically stirred at 1000 rpm for 30 min to prepare a uniform MMT dispersion with a concentration of 0.2 g mL^−1^. Finally, the resultant MMT dispersion (1 mL) was mixed with the as-prepared ANFs suspension (25 mL), thereby obtaining ANFs/MMT spinning dopes for wet spinning.

### Preparation of *n*-MXene and *p*-MXene/SWCNT-COOH Spinning Dopes

Ti_3_C_2_T_*x*_ MXene nanosheets were synthesized by selective removal of Al layers from the precursor Ti_3_AlC_2_ in the LiF/HCl etching solution as reported in previous publications [48–50]. Briefly, Ti_3_AlC_2_ powder (1 g) was slowly added to a Teflon container with 20 mL of HCl (12 mol L^−1^) and 1.6 g of LiF and magnetically stirred for 36 h at ambient temperature. Afterward, the resultant was repeatedly washed with distilled water and ultrasonicated for 3 h to obtain the monolayer of MXene nanosheets. In the end, the above MXene nanosheets were added into sericin/H_2_O solution (0.02 g mL^−1^, 5 mL) and mechanically stirred for 30 min, forming a uniform MXene dispersion (50 mg mL^−1^) as the spinning dope of *n*-segment TE fibers.

To acquire the *p*-type spinning dopes, 0.1 g of SWCNT was added into the mixture of sulfuric acid and nitric acid (3:1, v/v) and continuously stirred for 48 h. Subsequently, the acid-treated SWCNT (SWCNT-COOH) was repeatedly washed with deionized water through centrifugation at 8000 rpm to obtain a homogenous suspension (50 mg mL^−1^). Finally, the as-prepared suspension of MXene and SWCNT-COOH was mixed with a mass ratio of 1:1 as the spinning dope for wet spinning of *p*-segment TE fibers.

### Coaxial Wet Spinning of *p*–*n* Segment Core–Shell Fiber

*P*–*n* segment core–shell TE fibers constructed by the above as-prepared spinning dopes were fabricated through a continuously alternate coaxial wet-spinning process. The coaxial needle is composed of an outer needle (15 G, internal diameter: 1.4 mm) and an inner needle (22 G, internal diameter: 0.4 mm). The ANFs/MMT spinning dopes were injected into the outer needle, while the MXene and MXene/SWCNT-COOH spinning dopes were alternately injected into inner needles by using separate syringe pumps. The shell and core spinning dopes were extruded into an aqueous coagulation bath with 2 wt% of ammonium chloride (NH_4_Cl) at speeds of 300 and 150 μL min^−1^, respectively. The constant stretching ratio during the coaxial spinning was approximately 1.1. Then, the above resultant fibers were soaked in 50% tert-butyl alcohol aqueous solution to remove the part of the water. Subsequently, the fibers were obtained by gather them onto a winding spool and freeze-dried to obtain *p*–*n* segment core–shell fibers. The length of *p*–*n* repeat unit of the TE fiber was 1.2 cm, and an electrical interconnection (Ag NWs electrode) between the two doped sections was 2 mm.

### Characterization

The microstructural morphology and X-ray spectroscopy (EDX) elemental mappings of *p*–*n* segment TE fibers were characterized by scanning electron microscopy (SEM, E1856-C2B, USA). Morphologies of ANFs dispersion were observed with a transmission electron microscope (TEM, JEOL JEM F200, Japan). A Fourier infrared spectrometer (FTIR, TENSOR-27, Germany) was employed to characterize the functional groups. The crystalline structures of samples were measured using X-ray diffraction patterns (XRD, D8 Advance, Germany). X-ray photoemission spectroscopy (XPS, Thermo Scientific K-Alpha) was tested to analyze the surface elements and binding energies. The electrical properties of fibers were estimated on a four-probe resistivity meter (RTS-11, China). Mechanical properties tests were performed on a tensile testing machine (Instron 3365, USA) at a loading rate of 5 mm min^−1^. The pore size distribution and surface area of the aerogel fibers were analyzed by using Barrett–Joyner–Halenda (BJH) and Brunauer–Emmett–Teller (BET, Tristar II, USA) methods. The thermal decomposition behavior of fibers was estimated through a thermogravimetric analyzer (TGA, PerkinElmer STA 6000, USA) at a scanning rate of 10 °C min^−1^ in a nitrogen atmosphere. The combustion behaviors of fibers were further characterized using a micro calorimeter (FTT FAA-PCFC, UK). The flame retardancy of fiber was measured through limiting oxygen index test (LOI, repeated 5 times, Tech-GBT2406-1). The power factor, Seebeck coefficient (S) and electrical conductivity were simultaneously studied by a Seebeck coefficient and resistivity test system. The millivolt voltmeter (Dongguan Daxian Instrument, China) was applied to record the output voltage signal generated by TET.

## Results and Discussion

### Coaxial Wet Spinning of *p*–*n* Segment Core–Shell TE Fibers

**Fig. 1:**
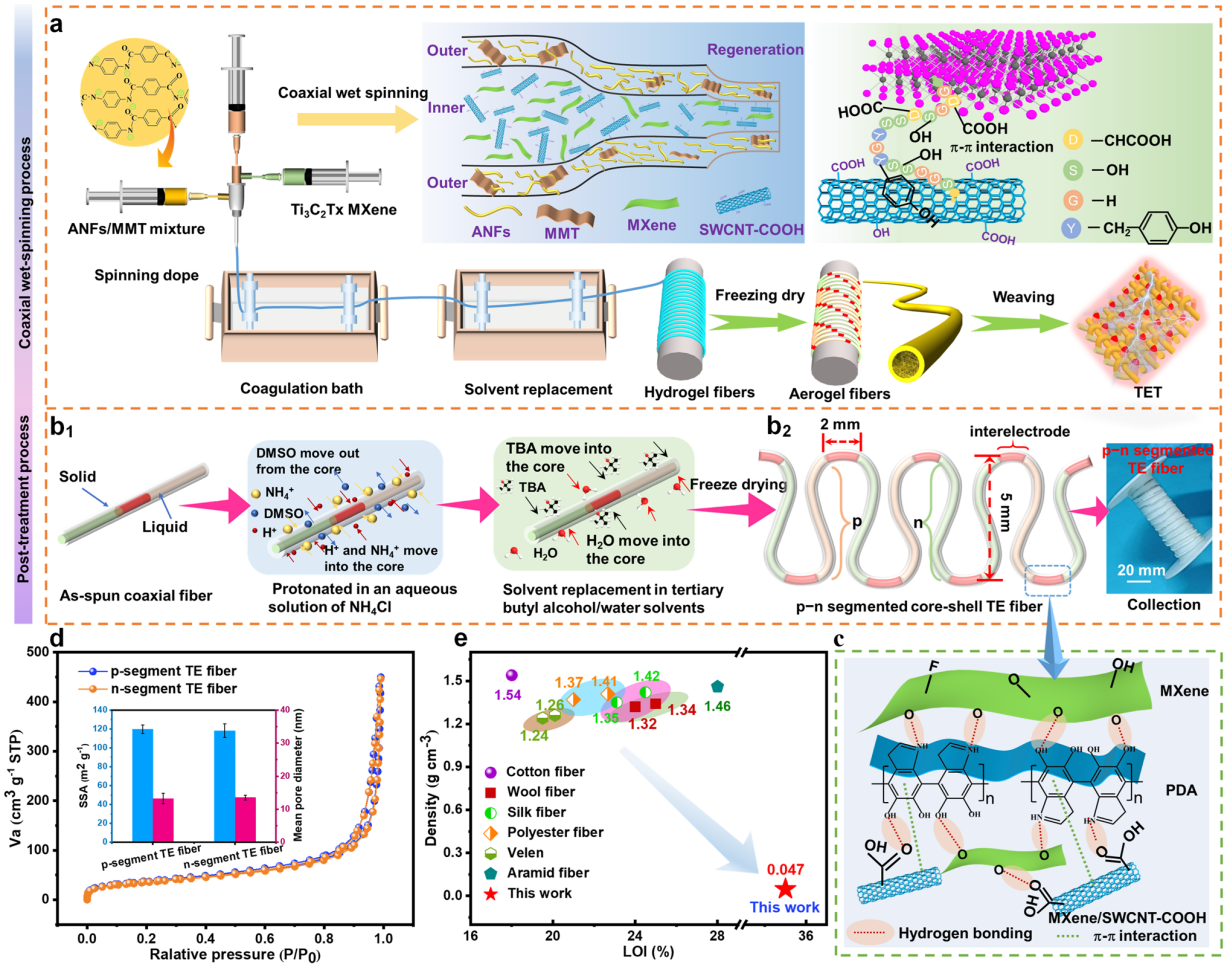
Coaxial wet spinning of *p*–*n* segment core–shell TE fibers. **a**, **b** Schematic of the coaxial wet-spinning and post-treatment process for producing core–shell TE fibers. **c** The formation of hydrogen bonding and *π*–*π* interaction among MXene, PDA and SWCNT-COOH. **d** Nitrogen adsorption/desorption isotherm of *p*-segment and *n*-segment TE fibers. The inset displays the specific surface area (SSA) and mean pore diameter. **e** Comparison of density and LOI between the as-prepared *p*–*n* segment core–shell TE fibers and commercial fibers

The core–shell Ti_3_C_2_T_*x*_ MXene-based *p*–*n* segment TE fibers were made through continuously alternate coaxial wet-spinning approach (Fig. 1a). Firstly, the MXene nanosheets were typically prepared by selectively removing the Al layers from the precursor Ti_3_AlC_2_ in the LiF/HCl etching solution and then ultrasonic exfoliation (Fig. S1a–d). As shown in Fig. S1e, the (002) diffraction peak of MXene displays a lower angle (5.02°) than that of Ti_3_AlC_2_ (9.56°), demonstrating the successful production of the MXene. Besides, the crystal planes of (004), (101) and (104) disappeared completely after the etching and delamination process. Subsequently, the *n*-type spinning dopes as core materials were obtained via mixing MXene nanosheets and sericin/H_2_O solutions after magnetic stirring. Sericin is a natural macromolecule composed of amino acid residues connected by peptide bonds, which contained approximately 70% hydrophilic residues that have strong *π*–*π* interactions with the MXene surface in *n*-type spinning dopes (Fig. S2) [51]. The sericin-treated MXene will possess abundant hydroxyl group on the surface of MXene, which provides abundant active sites for the formation of hydrogen bond cross-sectional interaction, and promotes the stability of MXene dispersion. Thereafter, the *n*-type spinning dopes were mixed with 50 mg mL^−1^ SWCNT-COOH dispersion in a mass ratio of 1:1 to acquire the *p*-type spinning dopes as another core materials. Among them, the SWCNT-COOH was fabricated by oxidation in a H_2_SO_4_/HNO_3_ mixed acidic solution, which could effectively improve the conductivity and dispersibility of SWCNT-COOH according to the removal of the external defect layers on the SWCNT surface (Fig. S3). As shown in Fig. S4, the FTIR curve of SWCNT-COOH showed obvious characteristic peaks at 3442, 1641 and 1390 cm^−1^, corresponding to –OH, C=O and –NO_2_ groups, indicating that carboxyl groups were produced in the carbon tube after acid treatment of SWCNT-COOH.

Subsequently, MMT was introduced into aramid nanofibers as shell spinning dopes to enhance the protection of TE core materials. Firstly, a stable dark red ANFs solution was formed from PPTA (average diameter and length of 13 μm and 7 mm, respectively) by deprotonation of the macroscopic aramid thread in KOH/H_2_O/dimethyl sulfoxide (DMSO) system (Figs. S5–S8). TEM images exhibited that the prepared ANFs dispersion possesses a radial size of ∼ 10 nm, a length of several micrometers with a large aspect ratio (Fig. S9). Figure S10 shows that the ANFs aerogel fiber and PPTA fibers displayed similar FTIR and XRD patterns, indicating that the structure of the nanofibers remained constant during the dissolution. Secondly, MMT was uniformly dispersed in the ANFs spinning dopes and provided flame-retardant protective shell for the *p*–*n* segment TE fibers. With the above as-prepared spinning dopes, *n*-type MXene or *p*-type MXene/SWCNT-COOH and ANFs/MMT spinning dopes were injected into the inner and outer Y-shaped coaxial needle (connecting a two-head converter) by separated syringes pumps. Subsequently, the spun fibers are coagulated in an aqueous solution of NH_4_Cl (2 wt%), where ammonium ions can effectively cross-link negatively charged MXene nanosheets through electrostatic interaction, and the aramid chains were protonated by the water molecules (Figs. 1b1 and S11). Afterward, the water in the resultant hydrogel fiber was partially substituted through solvent exchange with a tert-butanol/water solution to inhibit the growth of ice crystals in the next freeze-drying process. Finally, the alternating *p*–*n* segment core–shell TE fiber was obtained with two pairs of *p*–*n* segments in 3 cm and then collected on reels (fiber diameter: 0.85 mm; linear density: 22.3 tex) (Fig. 1b2). The *n*- and *p*-type segments were electrically connected in series along the TE fibers, which are separated by silver nanowires (Ag NWs)/polydopamine (PDA) mixture as *p*–*n* interelectrode (2 mm). PDA can self-polymerize on the surface of Ag NWs and promoted the connection among Ag NWs, *n*-type MXene and *p*-type MXene/SWCNT-COOH through hydrogen bonding and *π*–*π* interaction forces (Fig. 1c).

The N_2_ adsorption–desorption was used to evaluate pore size distribution and specific surface area of the as-prepared *p*–*n* segment TE aerogel fibers. As shown in Fig. 1d, the isotherm of TE aerogel fibers exhibited a hysteresis loop at a relative pressure of 0.5–1 corresponding to typical type IV curves, reflecting the existence of a large quantity of mesoporous structure. Moreover, the specific surface area of *p*-segment and *n*-segment TE fibers acquired from Brunauer–Emmett–teller was approximately 119.78 and 118.04 m^2^ g^−1^, while the mean pore diameter was 13.237 and 13.515 nm, respectively. To highlight the superiority of the as-prepared *p*–*n* segment TE aerogel fibers to traditional fibers (wool, cotton, silk, ramie, viscose and velen), the fiber density and limiting oxygen index (LOI) are compared in Fig. 1e. The results showed that the *p*–*n* segment TE aerogel fiber displayed the lowest density (0.047 g cm^−3^) and the highest value of LOI (~ 35%) among these fibers. Therefore, the as-prepared *p*–*n* segment TE aerogel fiber exhibited potential application prospects for production of wearable fire warning sensors due to its unique advantage in terms of lightweight, fire resistance and excellent wearable on dynamic body curves.

### Morphology and Structure of *p*–*n* Segment Core–Shell TE Fibers

**Fig. 2:**
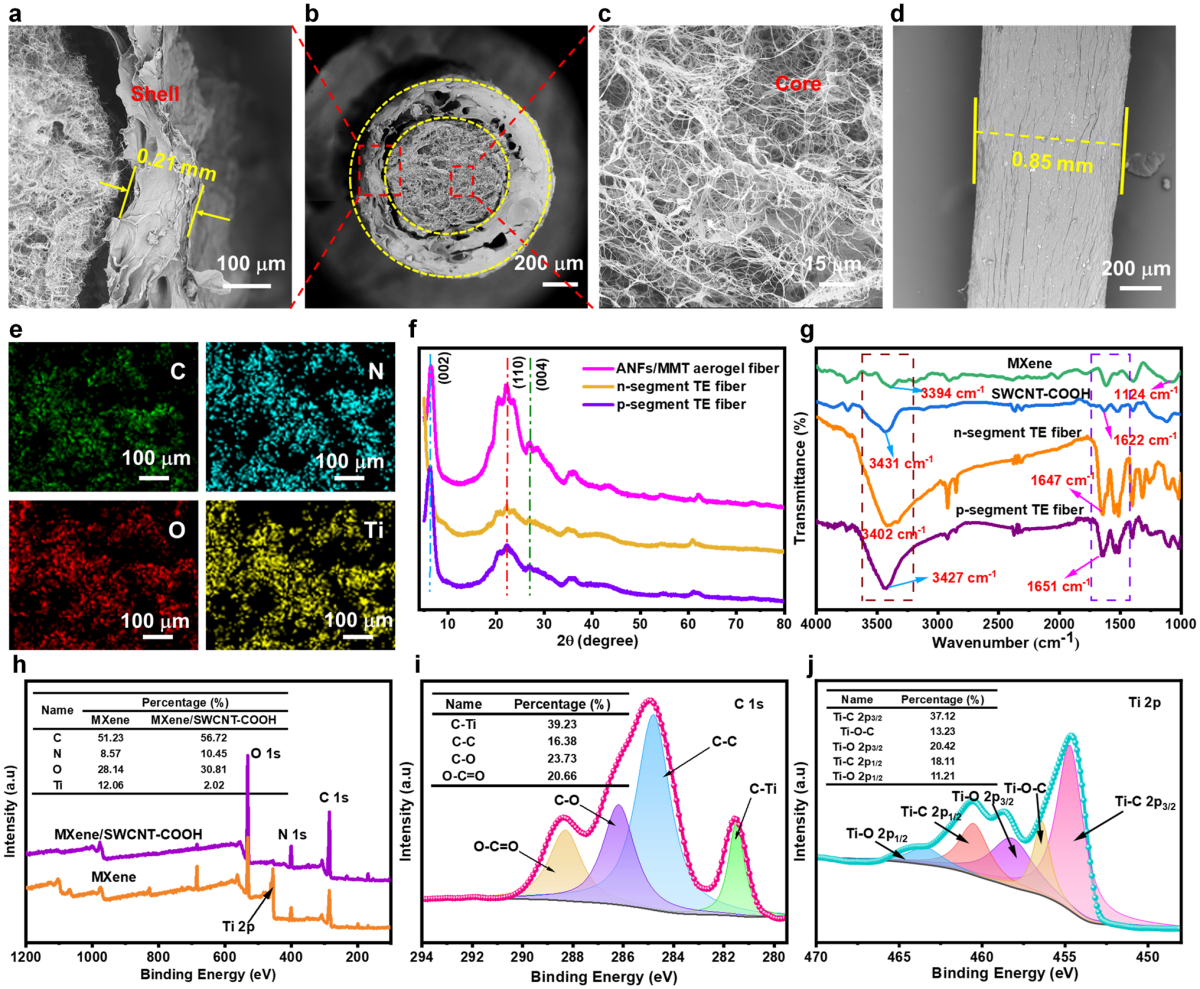
Microstructural characterizations of *p*–*n* segment core–shell TE fibers. **a** Cross-sectional SEM image of TE fiber shell. **b** Cross-sectional SEM image of the *p*–*n* segment TE fibers. **c** SEM image of *p*-segment TE fiber core material. **d** Surface morphology of *p*–*n* segment TE fiber. **e** EDX mapping images of *p*-type core material in TE fiber. **f** XRD patterns of pure ANFs/MMT fiber, *p*-segment and *n*-segment TE fibers. **g** FTIR spectra of MXene, SWCNT-COOH, *p*-segment TE and *n*-segment TE fibers. **h** Wide-scan XPS spectra of *n*-type MXene and *p*-type MXene/SWCNT-COOH samples. High-resolution XPS spectra of **i** C 1*s* and **j** Ti 2*p* for MXene/SWCNT-COOH sample

The cross-sectional SEM images of *p*–*n* segment core–shell TE fiber displayed a regular concentric circle structure, indicating the formation of internal TE material and continuous protective sleeve (Fig. 2a). SEM images manifested that the outer diameter of *p*–*n* segment core–shell TE fiber was 853.12 ± 18.37 μm with a wall thickness of 211 ± 21.39 μm. Moreover, it was also observed that the core and shell were continuous and tightly fused without obvious gaps and holes (Fig. 2b). As shown in Fig. 2c, the SWCNT-COOH was presented in the “spider web” morphology in *p*-type MXene/SWCNT-COOH core material, indicating good self-assembly compatibility between the SWCNT-COOH and MXene. Similar to the pure ANFs fiber, the surface of alternating *p*–*n* segment TE fiber showed a dense solid shell and numerous wrinkles that grow along axial direction of the fiber (Fig. 2d). EDX elemental mapping revealed that *p*-type core material in the alternating *p*/*n*-type TE fiber showed the uniform distributions of C, N, O and Ti elements, proving that MXene and SWCNT-COOH were uniformly dispersed (Fig. 2e).

The XRD patterns of fibers were examined to evaluate the effects of the TE material on crystalline structure. As presented in Fig. 2f, the *p*–*n* segment TE fiber and the ANFs/MMT aerogel fiber without TE core materials all presented typical peaks of ANFs at ~ 6.12°, ~ 21.98° and ~ 26.94° corresponding to the (002), (110) and (004) planes. The above results implied that the addition of TE as core material in *p*–*n* segment TE fiber did not destroy the crystalline structure of ANFs/MMT shell. FTIR was applied to investigate interfacial interaction between core and the shell layers. Compared to pure ANFs aerogel fiber, the shell component in *p*–*n* segment TE aerogel fiber displayed the characteristic peaks of aramid, including the carbonyl group (C=O) stretching vibration at 1627 cm^−1^, the stretching vibration and deformation of N–H at 3427 and 1542 cm^−1^ and Ph–N vibration at 1406 cm^−1^ (Figs. 2g and S10a). As shown in Fig. 2g, the FTIR spectra of MXene show two characteristic absorption bands at 3394 and 1124 cm^−1^ corresponding to the vibration absorption of the hydroxyl group (–OH) and C–O. For the SWCNT-COOH, characteristic peaks were observed at 1622 and 3431 cm^−1^ corresponding to the C=O stretching vibration and –OH. Compared to the above-mentioned characteristic peak of MXene, SWCNT-COOH and ANFs, the prepared *p*-segment TE fiber did not appear new characteristic bands, indicating that no new chemical bonds were formed in *p*–*n* segment TE fiber. However, the characteristic absorption bands of C=O for the *p*-segment TE fiber shifted from 1627 to 1651 cm^−1^ compared with the original pure ANFs. This finding may be explained by the formation of intermolecular hydrogen bonds among the functional groups of the MXene, SWCNT-COOH and C=O group of ANFs.

XPS was further carried out to analyze chemical bonding between MXene and SWCNT-COOH, as shown in Fig. 2h. The survey spectrum of MXene/SWCNT-COOH exhibited C 1*s*, O 1*s*, Ti 2*p* and F 1*s* peaks located at 285.08, 531.02, 455.03 and 684.12 eV, respectively, verifying the existence of MXene in *p*-type TE core material. Several peaks of C 1*s* spectrum were displayed at 281.48, 284.78, 286.15 and 288.26 eV, attributing to C–Ti, C–C, C–O and O–C=O, respectively (Fig. 2i). Meanwhile, the peak in the Ti 2*p* spectrum could be divided into five peaks, namely Ti–C 2*p*_3/2_ (454.68 eV), Ti–O–C (456.38 eV), Ti–O 2*p*_3/2_ (458.11 eV), Ti–C 2*p*_1/2_ (460.56 eV) and Ti–O 2*p*_1/2_ (463.45 eV) for MXene/SWCNT-COOH (Fig. 2j). Notably, the presence of Ti–O–C at 456.38 eV confirmed the establishment of a strong Ti–O–C covalent bonding between MXene and SWCNT-COOH. The above-mentioned changes suggested the existence of cooperation between MXene and SWCNT-COOH, which was beneficial to the internal charge transfer in core–shell TE fiber.

### Mechanical and Thermal Decomposition Behaviors of *p*–*n* Segment Core–Shell TE Fibers

**Fig. 3:**
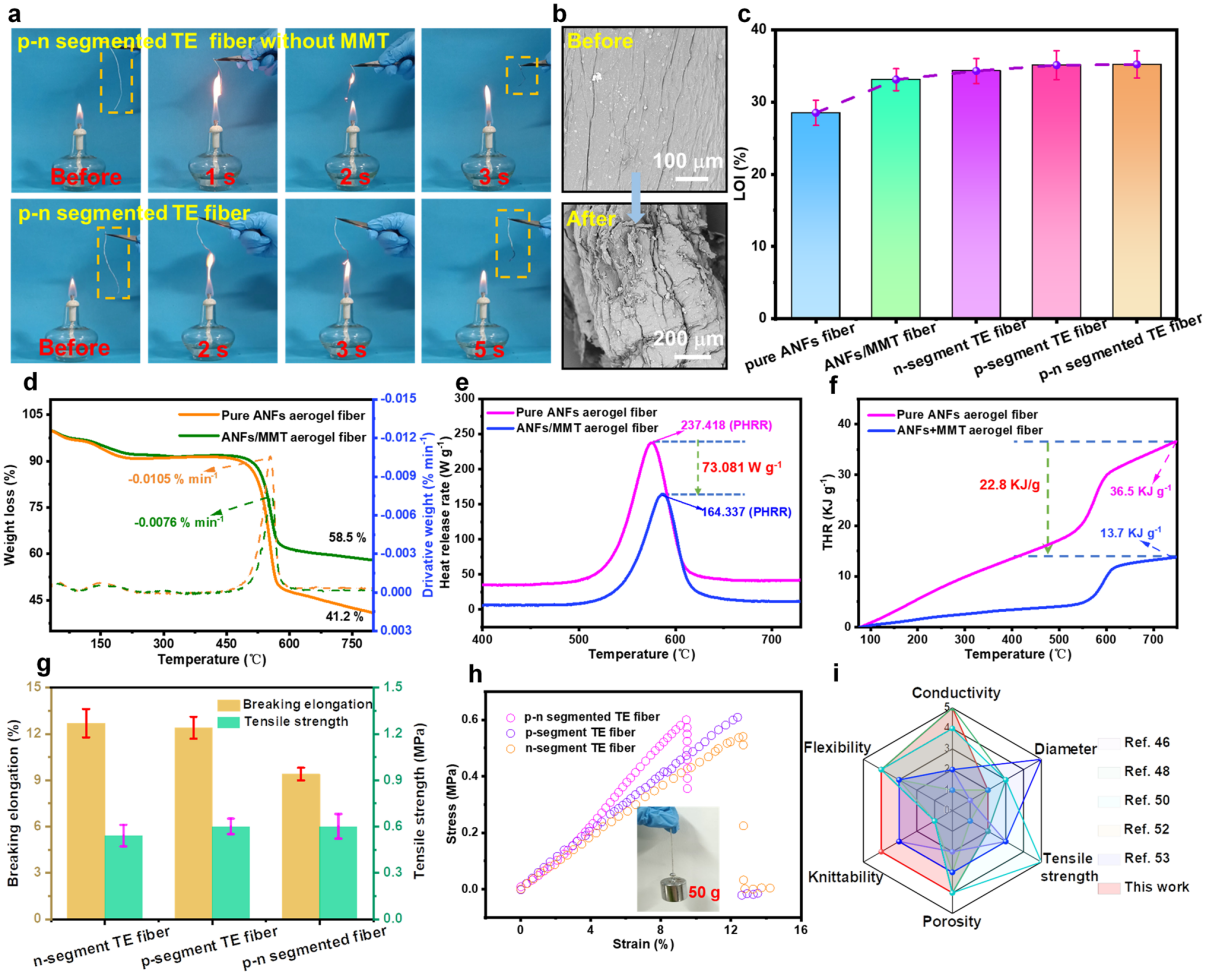
Flame retardancy and mechanical property of *p*–*n* segmented core–shell TE fibers. **a** Flame-retardant performance of *p*–*n* segmented TE fiber with and without MMT, respectively. **b** SEM images of *p*–*n* segmented TE fiber before and after being exposed to flame for 3 s. **c** LOI values of pure ANFs aerogel fiber, ANFs/MMT aerogel fiber, *n*-segment, *p*-segment and *p*–*n* segmented TE fibers. **d** TG and DTG curves of pure ANFs aerogel fiber and ANFs/MMT aerogel fiber in a nitrogen atmosphere. **e** HRR and **f** THR curves of pure ANFs aerogel fiber and ANFs/MMT aerogel fiber. **g** Mechanical properties and **h** typical stress–strain curves of *n*-segment, *p*-segment and *p*–*n* segmented TE fibers. **i** Diagram of typical properties (including conductivity, diameter, tensile strength, porosity, knittability and flexibility) of *p*–*n* segmented TE fiber compared with the previously reported aerogel fiber [46, 48, 50, 52, 53]

On account of practical application scenario, the flame retardancy is an important index for the *p*–*n* segment TE fibers as fire warning materials when used in firefighting clothing. The vertical burning test was conducted to further visually evaluate the flame-retardant performance of alternating *p*–*n* segment TE fibers. The *p*–*n* segment TE fiber without MMT was immediately ignited once it was in contact with the flame and would burn out within 3 s, as shown in Fig. 3a. In contrary, *p*–*n* segmented TE fiber did not ignite and retained its shape after being exposed to flame for 5 s, indicating that the MMT was beneficial to enhance the flame retardancy of the *p*–*n* segmented TE fiber. Figure 3b shows SEM images of the surface morphological structure of ANFs/MMT aerogel fiber before and after burning, respectively. The linear structure of *p*–*n* segmented TE fiber remained intact after burning for 3 s. The high flame retardancy performance of ANFs/MMT shell effectively prevents the heat transfer and protects the TET core materials from oxidization. Furthermore, EDX spectra of the *p*–*n* segmented TE fiber before and after burning are shown in Figs. S12–S13. The results showed that the contents of the C and O elements increased compared with those of the unburned fiber. However, the Ti elements did not appear after being burned, indicating that the ANFs/MMT as a protective sleeve was effective in protecting the TE core material. In addition, the LOI is used to assess the combustion performance of the aerogel fibers. The MMT endowed the ANFs/MMT aerogel fiber with an LOI value of 33.1%. The LOI values increased by 16% compared with the pure ANF fiber, as shown in Fig. 3c. The LOI value was ~ 35% in the *p*- and *n*-segment TE fibers, exhibiting similar and outstanding flame retardancy properties.

TGA and DTG were applied to investigate the thermal stability of the ANFs/MMT shell and *n*/*p* TE core materials in the *p*–*n* segmented TE fiber, as shown in Figs. 3d and S14. In the nitrogen atmospheres, the residual mass and the maximum thermal weight loss rate of ANFs/MMT shell were 58.5% and 0.0076% min^−1^, suggesting that the incorporation of the MMT into *p*–*n* segmented TE fiber could enhance thermal stability by building a physical barrier in the shell. As presented in Fig. S14, the *n*-type MXene and *p*-type MXene/SWCNT TE core materials underwent a major degradation stage from 250 to 400 °C, which mainly corresponds to the thermal decomposition of sericin. The results also indicated that ANFs/MMT shell could effectively protect the integrity of the TE core materials before the thermal degradation of ANFs/MMT shell, which was attributed to the thermal decomposition temperature of the shell was higher than that of the TE core materials. Moreover, the micro-scale combustion calorimetry was employed to further study the combustion behavior of ANFs/MMT shell. The peak heat release rate (PHRR) and total heat release (THR) curves of pure ANFs aerogel fiber and ANFs/MMT aerogel fiber without TE core material are shown in Fig. 3e, f, respectively. The PHRR values of the pure ANFs aerogel fiber and ANFs/MMT aerogel fiber without TE core materials were 237.418 and 164.337 W g^−1^ and the THR values were 36.5 and 13.7 kJ g^−1^, respectively. It was found that the PHRR and THR of ANFs/MMT aerogel fiber were reduced by 30.78% and 62.47%, respectively, compared with the pure ANFs aerogel fiber.

Mechanical performance is an important parameter to ensure wearability of TET-based fire warning sensor when used in firefighting clothing. Figure 3g exhibits the typical tensile stress–strain curves of *p*-segment, *n*-segment and alternating *p*–*n* segmented TE fibers. The *p*-segment TE fiber and *n*-segment TE fiber showed the maximum elongations of 12.4% and 12.7% under the tensile stress values of 0.54 and 0.6 MPa in Fig. 3h, respectively. By contrast, the *p*–*n* segmented TE fiber showed a similar tensile strength of 0.56 MPa and a break elongation of 9.4%. The fracture mechanism of *p*–*n* segmented core–shell TE fiber was proposed based on the foregoing analysis. Initially, the crumpled ANFs and MXene nanosheets could be straightened by applying external forces, and their alignment and degree of orientation were steadily enhanced with the increase in the external force. The strong interfacial force between the ANFs shell and MXene core was conducive to block the relative sliding of the MXene core in *n*/*p*-segment, thus providing effective load transfer and dissipating large energy for *p*–*n* segmented TE fiber to fracture (Fig. S15). Moreover, the *p*–*n* segmented core–shell TE fiber (diameter: 0.85 mm) could withstand a weight of 50 g without breaking. When the aforementioned fiber was spirally twined on a pen and evenly knotted, it did not show any fracture, indicating good flexibility and practical mechanical strength (Fig. S16). Furthermore, the comprehensive property of *p*–*n* segmented core–shell TE fiber was further investigated by comparing with previously reported aerogel fiber, including conductivity, diameter, tensile strength, porosity, knittability and flexibility (Fig. 3i). In particular, each performance was divided into five grades, and the evaluation criteria for grade are shown in Table S1. Apparently, *p*–*n* segmented TE fibers demonstrated preferable performances in conductivity (5 grade), knittability (4 grade) and flexibility (4 grade), which guaranteed the sensibility and wearability of *p*–*n* segmented TE fibers-based fire warning electronics.

### TE Properties of Individual *p*-segment and *n*-segment TE Fibers

**Fig. 4:**
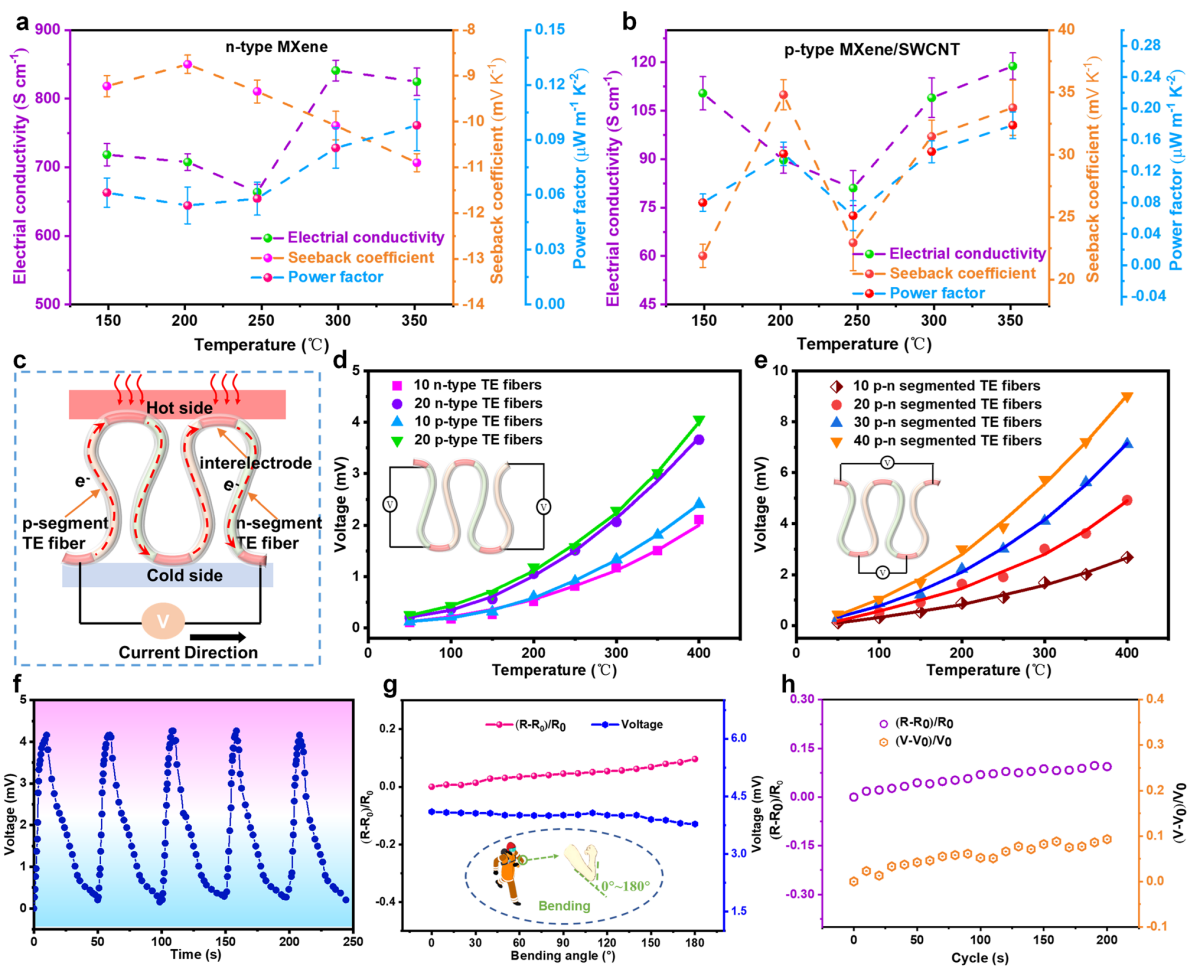
TE performance of *p*–*n* segmented core–shell TE fibers. TE properties of **a** *n*-type MXene and **b** *p*-type MXene/SWCNT-COOH materials, including power factor, Seebeck coefficient at different temperatures and electrical conductivity. **c** TE generation mechanism of *p*–*n* segmented TE fibers. **d** Output voltage of different numbers of *p* or *n*-type fibers at various temperature. **e** Open-circuit voltages of *p*–*n* segmented TE fibers including different pairs of *p*–*n* segments under different temperature. **f** Output voltage curves of *p*–*n* segmented TE fibers during alternating heating at 400 °C and cooling at room temperature for 5 cycles with 20 pairs of *p*–*n* segments. **g** Resistance changes and output voltage under different bending angles for TE fibers composed of 20 pairs of *p*–*n* segments. **h** The effect of bending cycles on resistance and output voltage at a bending angle of 150° with 20 pairs of *p*–*n* segments in TE fiber

The temperature-dependent TE properties of *n*-type MXene and *p*-type MXene/SWCNT-COOH materials are shown in Fig. 4a, b. As shown in Fig. 4a, the Seebeck coefficient of MXene was negative ranging from 150 to 350 °C, indicating that the internal charge carriers were mainly electrons. The absolute Seebeck coefficients of *n*-type MXene core material decreased at the beginning and then gradually increased, and the electrical conductivity exhibited an opposite trend with increasing temperature, displaying a typical metal-like behavior. With the addition of SWCNT-COOH in the MXene, the Seebeck coefficients changed to positive values in the range of 150–350 °C, confirming the *p*-type characteristics of carriers in MXene/SWCNT-COOH TE core materials (Fig. 4b). Compared to the Seebeck coefficient of MXene/SWCNT-COOH TE core materials, power factor showed a similar temperature dependent tendency. Meanwhile, the Seebeck coefficients of the MXene/SWCNT-COOH TE core material first increased with the temperature increase and then rapidly decreased when the temperature rose from 150 to 250 °C, followed by a slow increase again. Moreover, the electrical conductivity of *p*-type MXene/SWCNT-COOH TE core material first decreased, reaching the minimum value of 81.05 S cm^−1^ at 250 °C, and then increased in the subsequent temperature range. The electrical conductivity of *p*–*n* segmented TE fiber with two pairs of *p*–*n* segments was 23.76 S m^−1^ at room temperature.

The power generation mechanism of *p*–*n* segmented TE fibers is shown in Fig. 4c. The temperature difference was produced at the two sides of the *p*–*n* segmented TE fibers by heating bottom of the *p*–*n* junctions; therefore, the heat flow was transferred along the *p*–*n* segmented TE fibers from hot part to the cold part, resulting in a voltage generation. As presented in Fig. 4d, the number of fibers affect output voltage of *p*-type TE fiber and *n*-type TE fibers at different temperatures was analyzed, respectively. It was found that the output voltage increased approximately linearly with temperature, and the output voltage of *p*-type TE fibers (consisting of 20 filaments) was nearly twice that of the *p*-type TE fiber containing 10 filaments. Specifically, the output voltage of *p*-type fibers containing 10 filaments was 2.31 mV, and that of *p*-type TE fiber (consisting of 20 filaments) was 4.17 mV at the same temperature of 400 °C. Meanwhile, *p*-type TE fiber and *n*-type TE fiber with the same fiber amount displayed almost the same output voltage value, indicating that they can be matched to achieve electric series in the *p*–*n* segmented TE fiber. In addition, Fig. 4e displays the linearly increased output voltage with the number of *p*–*n* segmented TE fibers or increasing temperature. When *p*–*n* segmented TE fibers length increased from 10 to 40 pairs of *p*–*n* segment, the output voltage increased from 2.68 to 9.01 mV at the temperature of 400 °C, which conformed to the basic law of traditional TE materials.

To explore the repeatability and stability of power generation performance of *p*–*n* segmented fibers with 20 pairs of *p*–*n* segments, the TE fibers were subjected to five cycles of alternating heating (400 °C) and cooling (room temperature) (Fig. 4f). It was discovered that the highest output voltage of *p*–*n* segmented fibers with 20 pairs of *p*–*n* segments at 400 °C remained consistent at around 4.25 mV after five cycles of repeated heating and cooling. Considering that the *p*–*n* segment TE fiber-based self-powered fire warning electronics are inevitably bent during wearing, the resistance changes of (*R*-*R*_0_)/*R*_0_ (*R*_0_ and *R* are resistance before and after bending, respectively) and the output voltage were investigated under different bending angles from 0 to 180° at room temperature (Fig. 4g). The results indicated that the variation range of resistance was less than 9.6% and the maximum loss rate of output voltage was 7.8%. To further demonstrate the durability of the *p*–*n* segment TE fiber, the resistance changes of (*R*-*R*_0_)/*R*_0_ and the output voltage changes of (*V*-*V*_0_)/*V*_0_ (*V*_0_ and *V* are output voltage before and after bending, respectively) were measured after 200 cycles of 150° bending (Fig. 4h). The results revealed that the maximum fluctuation of voltage and resistance was 9.7% and 9.3% after 200 times bending-releasing circle, respectively. We suggested that the stable resistance and output voltage performance were mainly attributed to tough ANFs/MMT protective shell in *p*–*n* segment TE fiber. The above results revealed that the good flexibility and durability of *p*–*n* segment TE fiber would be an appealing prospect in preparation of flexible and stable TE fiber-based fire warning sensors.

### Structure and Electrical Transport in TET

**Fig. 5:**
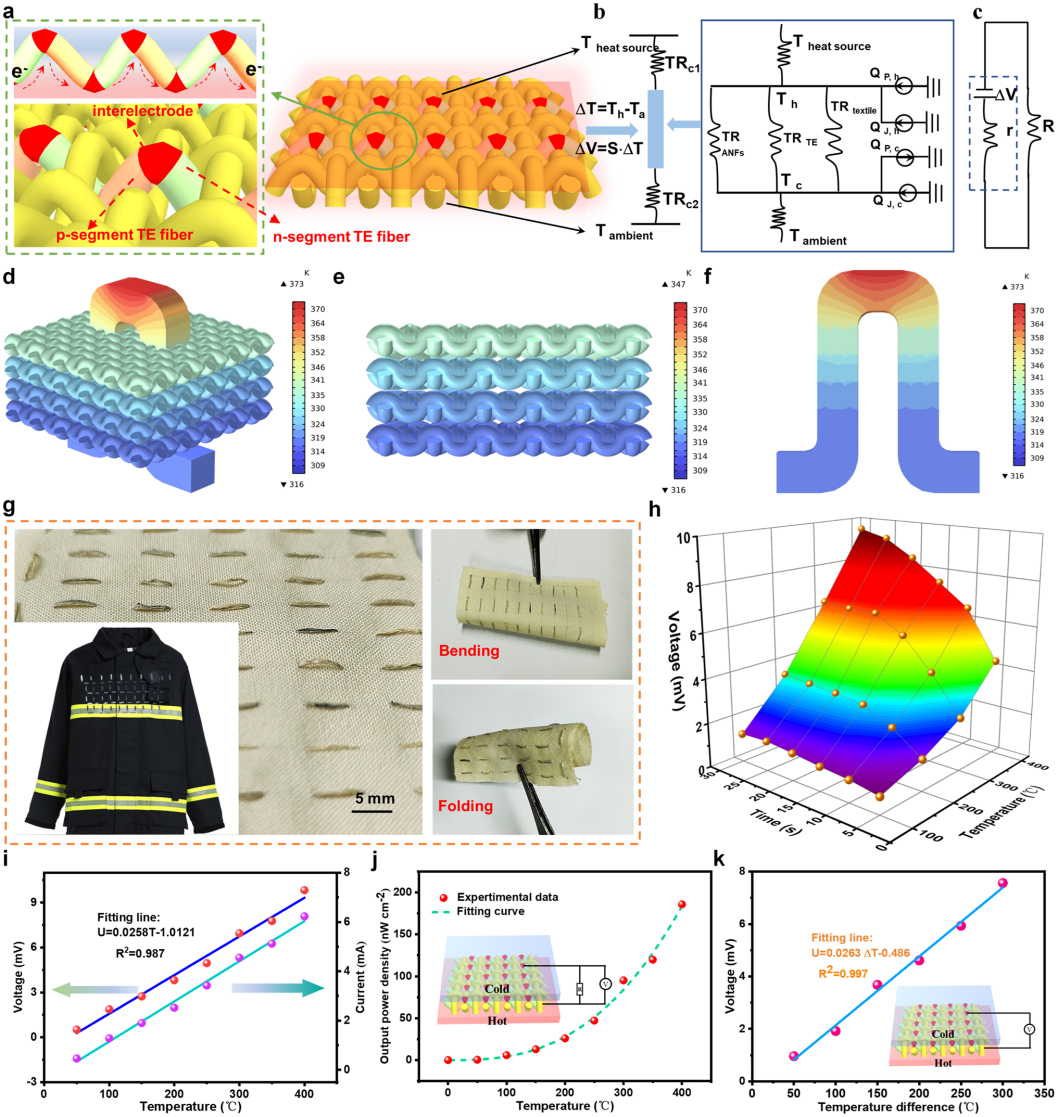
Electrical and thermal transport of *p*–*n* segmented TE fiber in TET. **a** Design structure of TET and *p*–*n* junction alternate between the hot and the cold sides. The illustration (left) is the TET’s cross section, confirming vertical arrangement of *n*-segment and *p*-segment in TE fibers. **b** Thermal diagram during heat transport in the prepared woven TET. Note: TR on behalf of thermal resistance (TR_c1_ and TR_c2_ are the thermal resistances of the cold and hot sides, respectively, and TR_ANFs_, TR _textile_ and TR_TE_ are thermal resistance of the hollow ANFs/MMT fiber layer, textile substrate and *p*–*n* segmented TE fiber, respectively). *Q* represents the generated heat inputs (*Q*_P, h_ and *Q*_J, h_ are input thermal produced via Peltier effect and Joule heating at the hot sides, respectively; *Q*_P, c_ and *Q*_J, c_ are input thermal produced via Peltier effect and Joule heating at the cold sides, respectively). **c** Circuit diagram of TET during electrical transmission in TET. Note: R and r are external and internal resistances of TET, respectively. **d** The temperature distribution of the TET by finite element analysis. The temperature distribution of the aramid fabric substrate **e** and *p*–*n* segmented TE fiber **f**. **g** Optical image of the resultant TET through stitching the alternating *p*–*n* segmented TE fiber into a flexible aramid fabric (5 cm × 4.5 cm), exhibiting outstanding flexibility, including bending and folding. **h** Output voltage curve of TET at the different heating temperatures ranging from 100 to 400 °C. **i** Open-circuit voltage and current as functions of temperature. **j** Maximum power density of as-prepared TET at various temperatures. **k** Output voltage curve of TET concerning temperature difference

With such alternating *p*–*n* segmented TE fibers, TET-based self-powered fire warning sensor (lateral size: 5 cm × 4.5 cm and thickness: 0.5 mm) with 50 *p*–*n* pairs and a density of 201.6 g m^−2^ were successfully fabricated through stitching the alternating *p*–*n* segmented TE fibers into aramid fabric (Fig. 5a). In this configuration, the lateral areal density of *p*–*n* pairs in TET was ~ 2.2 pairs per cm^2^, and the *p* or *n* segment satisfied continuous *p*–*n* junctions were alternately exposed to hot and cold sides. Consequently, the carriers in each TE units will be transferred along the *p*–*n* segmented TE fiber in the same direction, so that output voltage multiplication can be achieved. The electrical and thermal circuit diagram of TET is shown in Fig. 5b, c based on the composition of TET. In this work, the temperature in the fire field acts as the heat source; the cold part of TET is in contact with human skin, thus forming a temperature difference. When TET generated electricity in the actual work, heat flux Q transfers to the other side of TET through the surface of the heat source (environment), and then into the inner structure of TET (containing ANFs/MMT shell layer, *p*–*n* segments and aramid fabric) to the bottom part contact with human skin. Similar to commercial TE generators, *p*/*n* elements are thermally integrated in parallel and electrically connected in series. Using the data in Fig. 5d–f, we built a TET model to clearly comprehend the heat transport in TET by finite element analysis. The electrical, thermal and dimension performances of each constituent in TET model are not different from above TET with *p*–*n* segmented TE fibers. When the TET was attached to a heat source at 200 °C, the absorbed heat flows in parallel between the individual components of the TET and the remaining heat was dissipated to the surrounding environment through its cold side. More importantly, the designed TET-based fire warning sensor can be easily integrated into protective clothing thanks to its good dynamic surface conformability such as bending and folding (Fig. 5g).

The diagram with three-dimension curved surface was used to record the output voltage of the as-prepared TET under different temperature conditions. The maximum output voltage produced increased from 1.28 to 9.54 mV (increasing by 8.26 mV) as the heating temperature increased from 100 to 400 °C, as presented in Fig. 5h. In addition, the rate of voltage generation increased with the increase in treating temperature. This situation indicates that the TET becomes more sensitive to higher temperatures. The enhanced sensitivity is beneficial in effectively reducing the fire warning trigger time when applied in the field of fire warning. Moreover, the output voltage, current and output power density of as-prepared TET at different temperatures (ranging from 100 to 400 °C) are shown in Fig. 5i, j, respectively. When the temperature was applied across the surface of the TET, the output voltage and current are nearly linear with the temperature (up to 9.83 mV and 6.21 μA at 400 °C, respectively) (Fig. 5i). According to the output power calculation formula *P* = *U*^*2*^*R*/(*R* + *r*)^2^, the maximum output power will be obtained when the internal resistance *r* is equal to the external resistance *R.* The maximum power density calculated in Fig. 5j is used to divided the output power by the effective area of TET (lateral size: 5 cm × 4.5 cm) to more accurately evaluate the TE performance. The maximum power density of TET was 185.82 nW cm^−2^ at the temperature of 400 °C and was increased in quadratic relation with temperature. Figure 5k displays that the maximum output voltage of TET was 7.5 mV at the Δ*T* = 573 K, which was linear to the heating temperature difference, and its fitted function relation with linear fitting degree *R*^*2*^ of 0.997 was “*U*_max_ = 0.0263Δ*T-*0.4861.” Consequently, the surface temperature of the firefighting clothing can be accurately determined by analyzing the relationship between the output voltage and the temperature difference to determine its damage condition in fire cases. In addition, as a wearable electronic textile, washability is a crucial index to assess the durability of TET-based fire warning sensors. As shown in Fig. S17, the maximum output voltage error values of TET were 7.7% after 20 washing cycles, suggesting a certain extent output voltage accuracy and stability.

### Early Fire Warning Performance of TET-based Fire Alarm Device

**Fig. 6:**
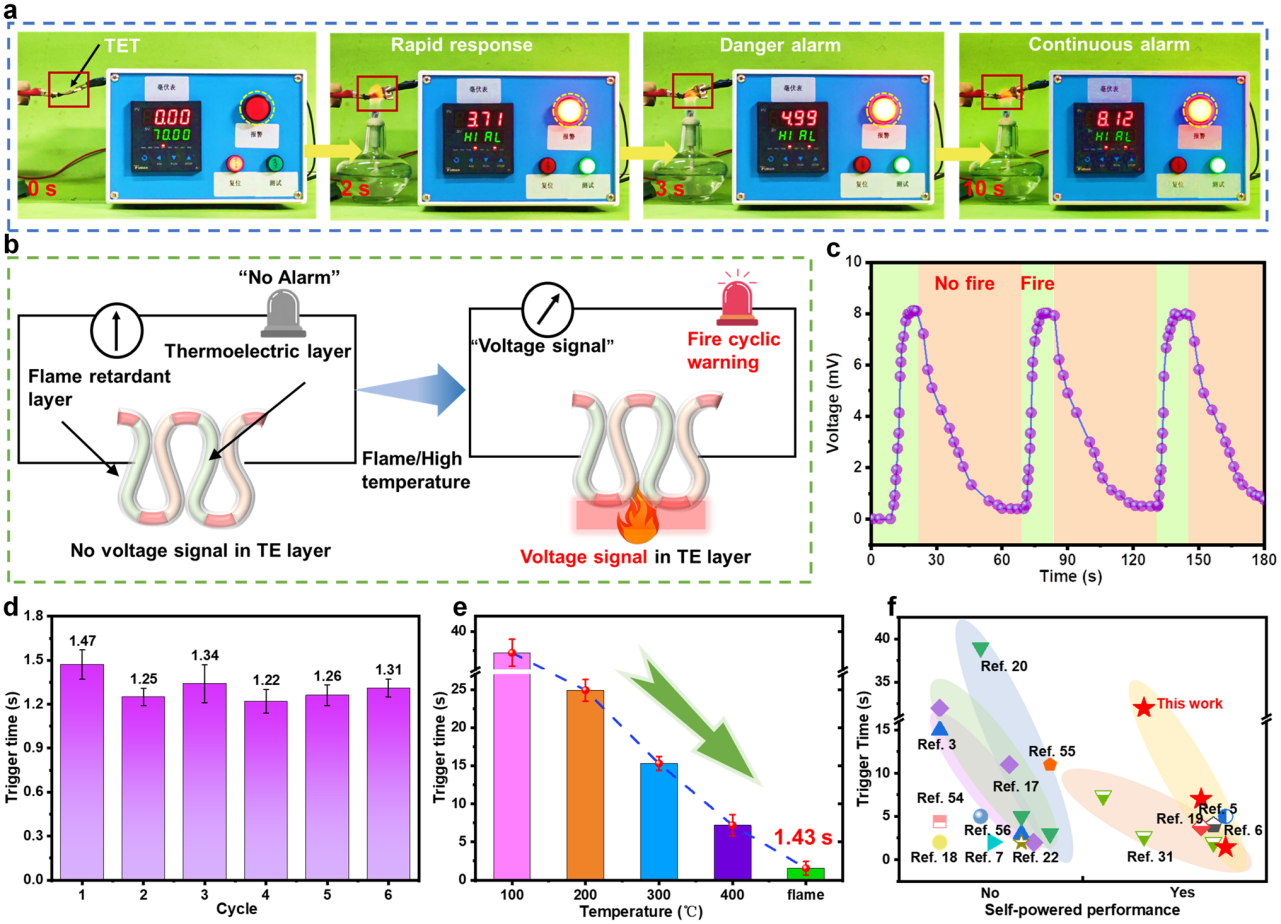
**a** Fire warning test for TET-based fire alarm device when exposed to alcohol lamp flame. **b** Schematic of early fire warning response mechanism. **c** Voltage curve of TET in three cyclic fire warning tests. **d** Trigger time of TET in fire alarm tests at 1–6 cycles. **e**. Fire warning trigger time of TET at various environmental temperatures. **f** Comparison of fire warning response time and self-powered warning performance of TET with other reported fire warning sensors [3, 5–7, 17–20, 22, 31, 54–56]

The TET could be used as a reliable and repeatable fire warning e-textile to detect the surface temperature of firefighting garments due to linear relationship between temperature and output voltage. In this study, the TET was connected to a millivolt voltage alarm using several wires, and the early fire warning system was constructed without need for an external power source (Fig. 6a). 1 mV was set as the early warning trigger voltage of TET. The corresponding warning process photos are shown in Fig. 6a to intuitively evaluate the fire warning behavior of TET. The TET could trigger the alarm within 2 s upon exposure to the alcohol lamp flame, which was a breakthrough advancement in sensitivity compared with the other commercial fire warning systems (~ 100 s) [1, 4]. The TET could generate voltage signals when exposed to temperature differences between the hot and the cold parts because of the thermoelectric characteristics of the TE materials, and it triggers the fire warning system once it detects high-temperature flame (Fig. 6b). Moreover, the TET can reactivate the fire warning system when it was once exposed to flames due to the reversible TE response characteristics of the TE material. This advantage provides a method to solve the problem that the GO-based fire warning sensor is not reusable. In Fig. 6c, the as-prepared TET was intermittently exposed to alcohol lamp flame at cycle periods of 60 s, and the output voltage was collected. The results manifested an average output voltage of 8.04 mV with a small standard deviation of 9.6%, suggesting the reliable repeatability of TET-based fire warning sensors. This feature was mainly attributed to the excellent thermal stability of the ANFs shell, which can efficiently protect the integrity of the core TE material structure. Figure S18 exhibits video screenshots of repeated fire warning test process for TET-based fire warning sensors. The results showed that the TET-based fire warning sensors could activate the fire alarm system in 1.66 s when it was encountered alcohol lamp flame once again (Video S1). Furthermore, the fire warning response times of the TET between the first and sixth were 1.47 and 1.31 s in six fire warning cycles, confirming the stable and long-lasting fire warning performance (Fig. 6d). Overall, the TET-based fire warning sensors exhibited sensitive warning response time and repeated fire warning capability under alcohol lamps, which could timely alert firefighter to evacuate to a safe position from the fire before the protective clothing malfunctioned on fireground.

Given that the response time of the early fire alarm has a strong correlation with the environmental temperature, the fire warning trigger time of the TET at various temperatures was tested (Fig. 6e). The trigger time of TET-based fire warning sensors displayed a significantly decrease with increasing temperature (for example, 37.2 s for 100 °C, 24.9 s for 200 °C, 15.3 s for 300 °C, 7.2 s for 400 °C and 1.43 s for alcohol lamp flame), demonstrating sensitive fire warning capability in high-temperature flame. We compared the fire warning response times and the power supply of fire warning under different high temperatures between our work and other various references based on the above-mentioned results of the TET-based fire warning sensors (Fig. 6f). According to the comparison result, the TET-based fire warning sensors showed a wide range of temperature sensing and ultrasensitive early fire warning performance without external power supply, which was superior to other fire warning sensors.

### Proof-of-Concept of Self-powered Fire Warning TET in Firefighting Clothing

**Fig. 7:**
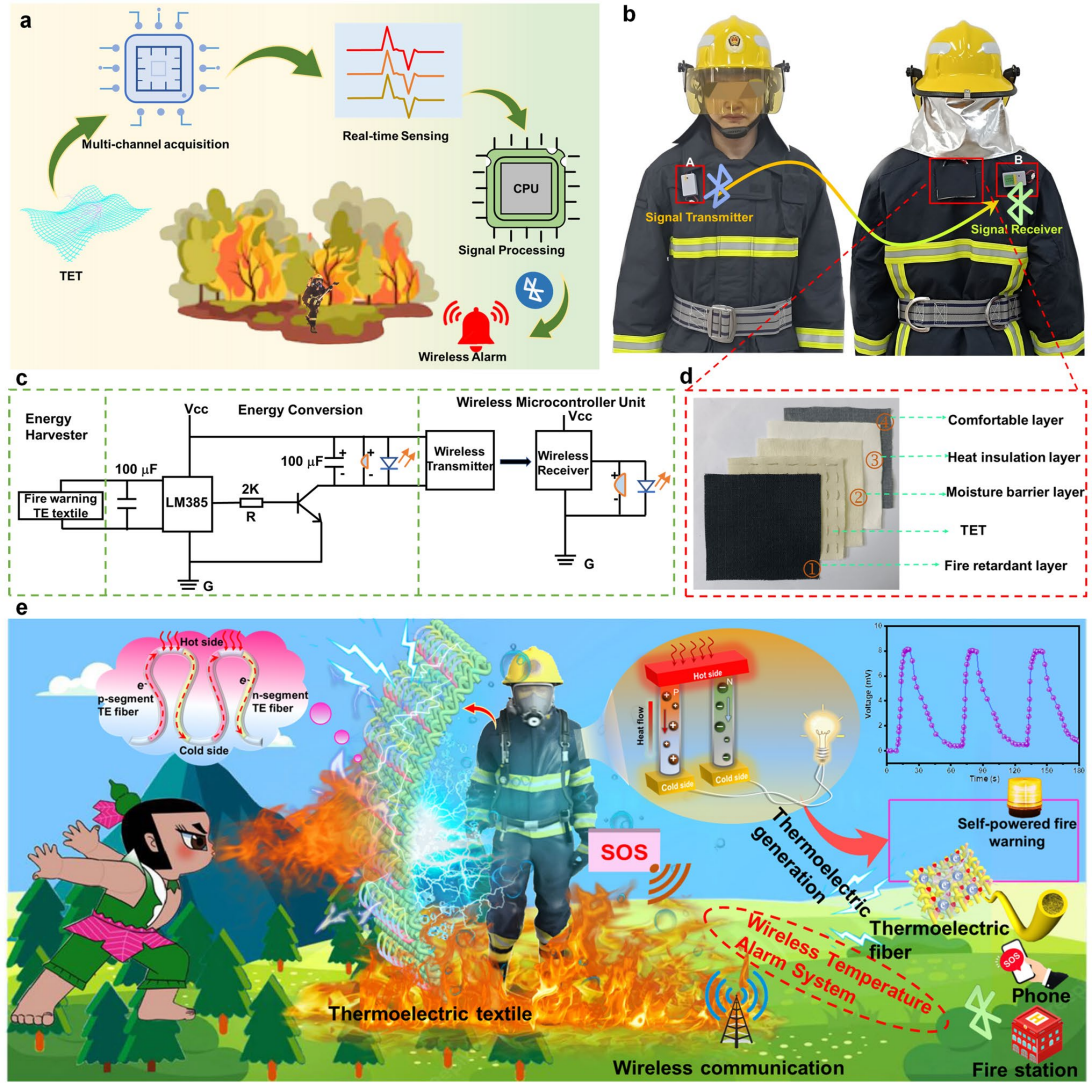
Application of TET in a self-powered wireless fire alarm system. **a** Schematic showing the TET as a self-powered fire warning sensor that provides active fire protection to firefighters. **b** Photographs of TET-based fire warning device that has been integrated into firefighting clothing. **c** Circuit diagram of TET-based self-powered wireless fire alarm system. **d** Integrated location of TET in firefighting clothing (including fire-retardant layer, TET, moisture barrier layer, heat insulation layer and comfortable layer). **e** Proof of concept of the self-powered fire warning TET in the firefighting clothing, with the TET serving as a fire warning sensor and a power supply and a wireless communication as a means of warning

To take full advantage of the TE properties of TET, we conceived a new wireless fire warning system to alert firefighters once the surface temperature of protective clothing closed to its own thermal decomposition temperature in a fire (Fig. 7a). The as-prepared TET was integrated between fire-retardant and moisture barrier layer on the back of firefighting clothing for energy conversion (Fig. 7b, d). The TET served as a self-powered signal conversion module to generate voltage signal when exposed to a high temperature to activate fire warning system without requiring additional power supply. Subsequently, the voltage signal was collected by the signal acquisition module of a single-chip microcomputer connected to the transmitter to reflect the temperature change. When the output voltage signal exceeds the trigger threshold (0.99 mV) for warnings, the wireless transmitter device will send a control command to the wireless receiver. Accordingly, an alarm in the form of light and ring is activated to promptly alert firefighters to evacuate. Figure 7c presents the logic design of the foregoing wireless warning system. The system was composed of three modules, including energy collection, energy transmission and a wireless microcontroller unit. We created a piece of true wireless detection equipment according to the above-mentioned concepts for verification. Moreover, we successfully tested this system with open flames indoors to confirm its usability in a fire (Figs. 7b, S19 and Video S2). The signal transmitter was integrated far from the signal receiver on the firefighting clothing, as shown in Fig. 7b. In the absence of flame, everything remains calm. However, an early warning system was immediately triggered as soon as flame was detected, activating an alarm to alert firefighters to safety evacuate. Figure 7e depicts the TET ultrasensitive temperature monitoring and self-powered fire warning performance. The integrated TET in firefighting clothing could detect the surface temperature of the suit and immediately alert firefighters to evacuate for their safety before the firefighting clothing malfunction. Alternatively, when the TET is exposed to flame or a high temperature, a wireless detection system can send a fire warning message to an operator who controls the status of these parameters from another remote location. The compatibility with body movement and sensitive fire warning performance of TET demonstrates the application prospect in firefighting clothing.

## Conclusions

In summary, temperature-arousing self-powered fire warning devices based on *p*–*n* segmented TE aerogel fibers were developed for providing active fire protection for firefighting clothing and ensuring the safety of firefighter during the firefighting operation and rescue mission. An alternating wet-spinning strategy was adopted to fabricate *p*/*n*-type alternating TE fibers with an outstanding electrical conductivity of 23.76 S m^−1^. Through periodically stitching of alternating *p*/*n*-type TE fibers into aramid fabric, TETs-based fire warning device containing 50 *p*–*n* pairs produced the open-circuit voltage of 7.56 mV with an output power density of 119.79 Nw cm^−2^ at a temperature difference of 300 °C. Consequently, the resulting voltage signals were converted into detected temperature based on a linear relationship between TE voltage and temperature. The result showed that the self-powered TETs-based fire warning device can be incorporated into firefighting clothing to achieve an accurate and repetitive temperature sensing at 100–400 °C. This integration enables timely alerted for firefighters to evacuate before firefighting clothing is burned by abnormally high-temperature flame. Moreover, the TETs-based fire warning electronics also exhibited outstanding flame retardancy (PHRR ~ 164.33 W g^−1^, LOI ~ 35%) and an ultra-low fiber density (0.047 g cm^−3^), ensuring the safety of a fire warning device in fire. The compatibility with body movement and breathability (air permeability ~ 114.45 mm s^−1^) of TET-based fire warning electronics demonstrate its potential application and open up opportunities for developing next generation wearable and self-powered fire warning device used in firefighting clothing.

### Supplementary Information

Below is the link to the electronic supplementary material.

Supplementary file1 (PDF 1486 KB)

Supplementary file 2 (MP4 18,684 KB)

Supplementary file 3 (MP4 13,642 KB)
