# Supplementary material for: Temperature-Arousing Self-Powered Fire Warning E-Textile Based on p–n Segment Coaxial Aerogel Fibers for Active Fire Protection in Firefighting Clothing
Source: Nanomicro Lett. 2023 Oct 13;15:226. doi: 10.1007/s40820-023-01200-8 (PMC10575845; doi:10.1007/s40820-023-01200-8)
Supplement: Supplementary file 1 — Supplementary file1 (PDF 1486 KB) [file 40820_2023_1200_MOESM1_ESM.pdf]

Supporting Information for

Temperature Arousing Self-Powered Fire Warning e-Textile Based on p-n Segmented Coaxial Aerogel Fibers for Active Fire Protection in Firefighting Clothing

Hualing He[1,2,#], Yi Qin[1,#], Zhenyu Zhu[1], Qing Jiang[1], Shengnan Ouyang[2], Yuhang Wan[1], Xueru Qu[1], Jie Xu[1], and Zhicai Yu[1,2,3,*]

[1] State Key Laboratory of New Textile Materials and Advanced Processing Technologies, School of Textile Science and Engineering, Wuhan Textile University, Wuhan 430200, People's Republic of China

[2] National Local Joint Laboratory for Advanced Textile Processing and Clean Production, Hubei Key Laboratory of Biomass Fibers and Eco-dyeing & Finishing, Wuhan Textile University, Wuhan 430200, People's Republic of China

[3] Jiangsu New Horizon Advanced Functional Fiber Innovation Center Co., Ltd., Suzhou 215000, People's Republic of China

[#] Hualing He and Yi Qin contributed equally to this work.

*Corresponding authors. E-mail: yuzhicaicai@163.com (Zhicai Yu)

S1 Experimental Section

S1.1 Synthesis of Ag NWs@PDA

Firstly, 50 mg Ag NWs were dispersed in 90 mL water and then stir magnetically for 10 minutes. Secondly, 0.18 g DA and 760 μL NaOH aqueous solution (2 mol/L) were added to the above mixture followed by stirring for 7 h at 60 °C to obtain AgNWs@polydopamine (PDA) spinning dope.

S2 Supplementary Figures and Tables

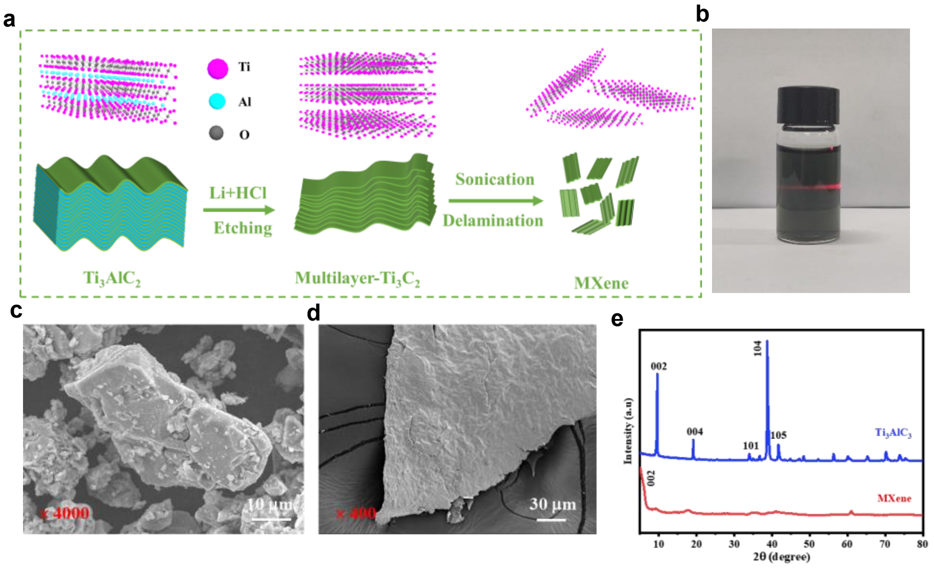

Fig. S1 a Fabrication schematic illustration of MXene nanosheets. **b** The digital image of MXene suspension. SEM images of **c** precursor Ti_3AlC_2 powder and **d** the MXene nanosheets with different magnifications. **e** X-ray diffraction patterns of Ti_3AlC_2 powder and MXene nanosheets

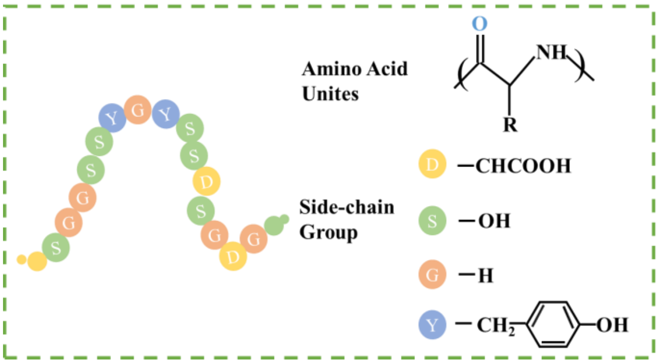

Fig. S2 Chemical structure of sericin

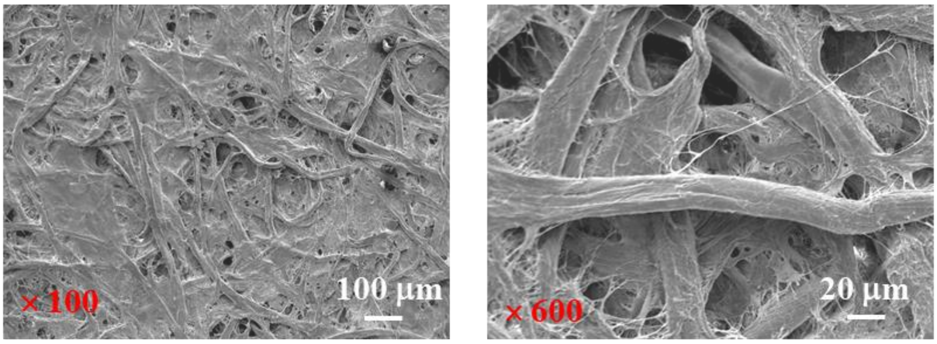

Fig. S3 SEM images of SWCNT-COOH with different magnifications

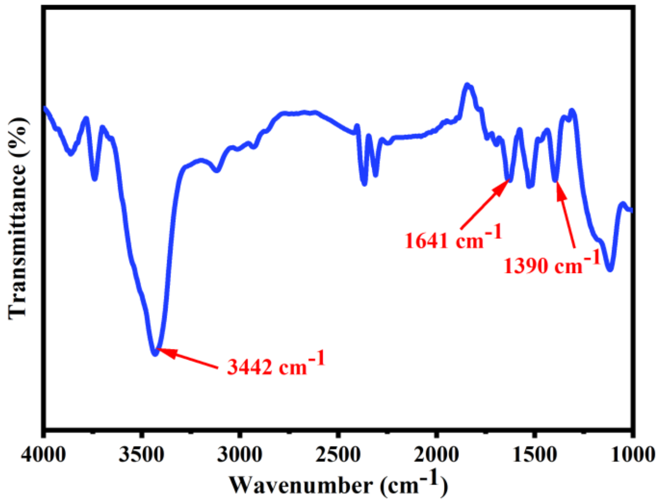

Fig. S4 FTIR spectra of SWCNT-COOH

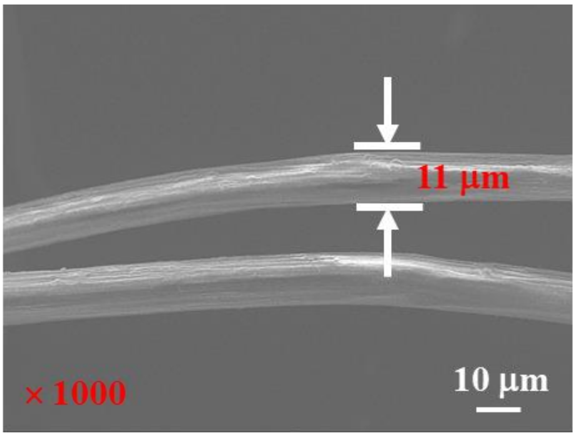

Fig. S5 SEM image of the surface morphology of commercial PPTA

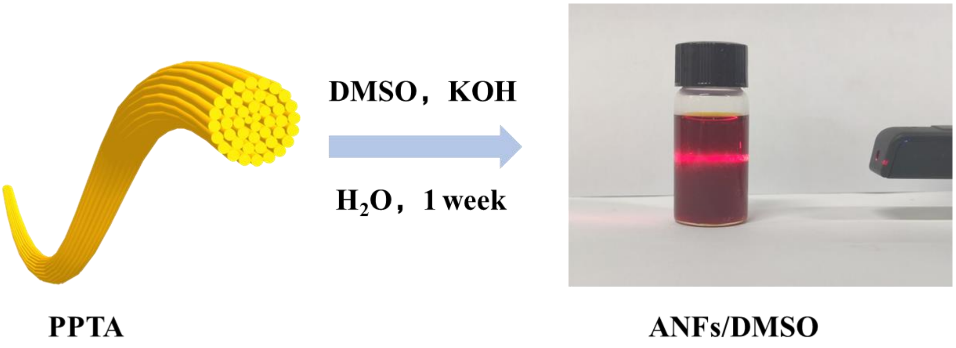

Fig. S6 Preparation process of the ANF dispersion

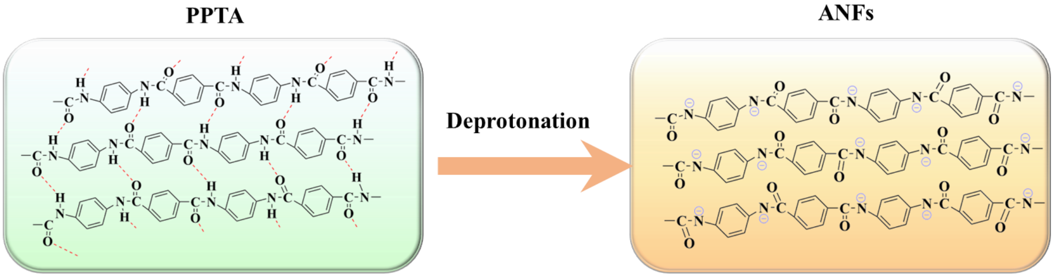

Fig. S7 Schematic diagram of PPTA fiber formation into ANFs dispersion in KOH/DMSO solution

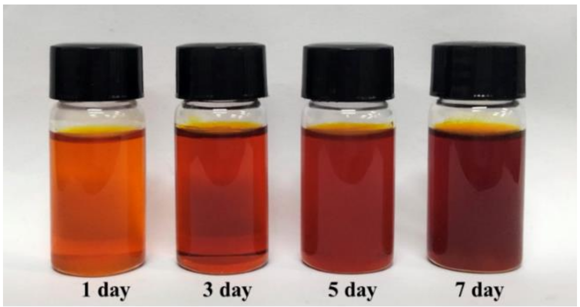

Fig. S8 Digital photos of ANFs/DMSO solutions at different dissolution times

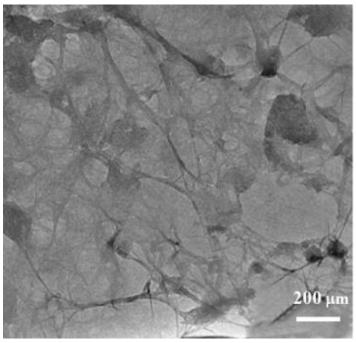

Fig. S9 TEM image of the as-prepared aramid nanofibers dispersion

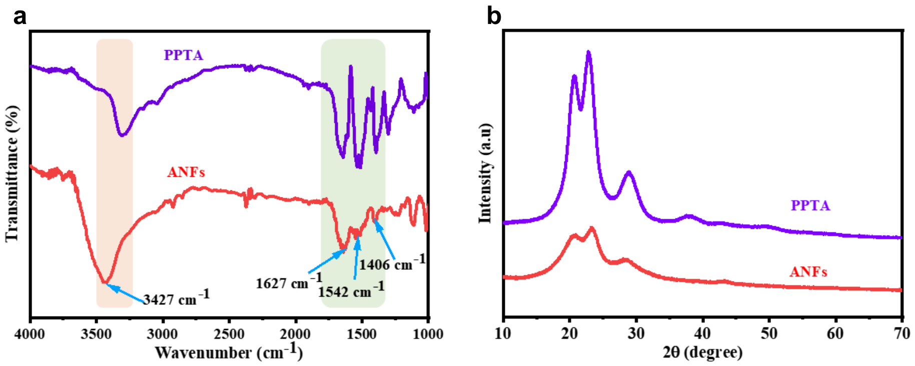

Fig. S10 a FTIR spectra and **b** XRD patterns of PPTA fibers before and after dissolution

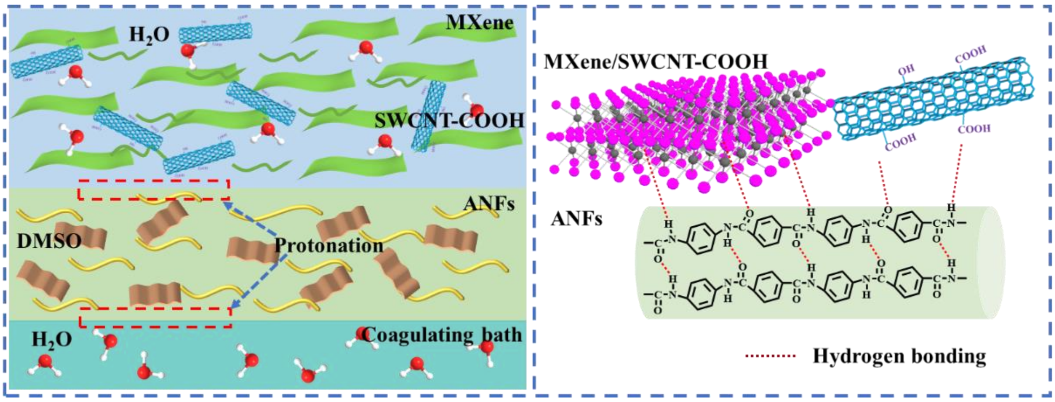

Fig. S11 Protonation process of ANFs, and the interfacial interactions among SWCNT-COOH, MXene and ANFs

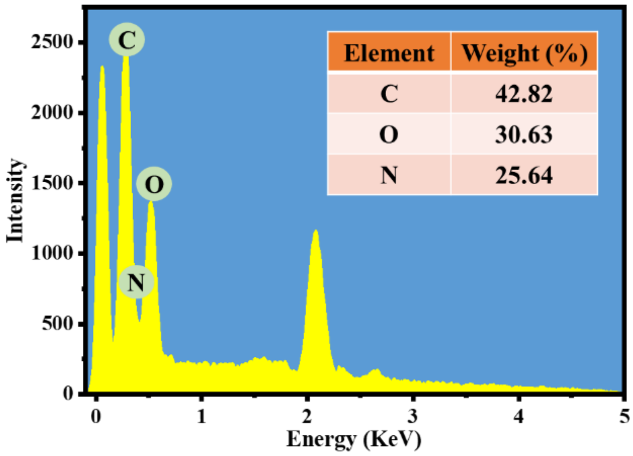

Fig. S12 EDX elemental mapping of p-n segmented TE fiber

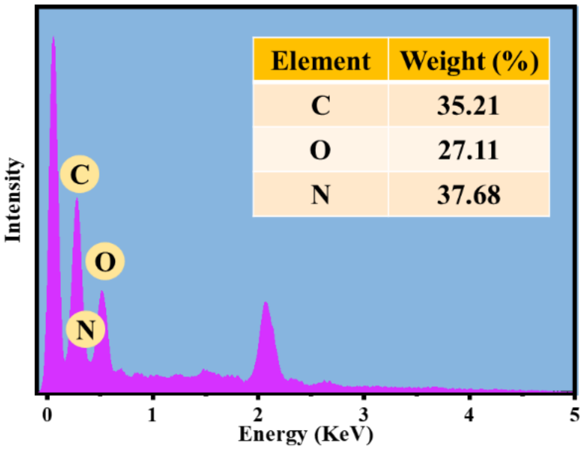

Fig. S13 EDX elemental mapping of p-n segmented TE fiber after being exposed to flame for 3 s

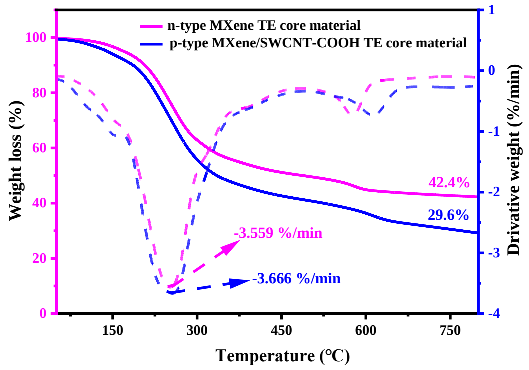

Fig. S14 TG and DTG curves of n-type MXene and p-type MXene/SWCNT-COOH TE core material in a nitrogen atmosphere

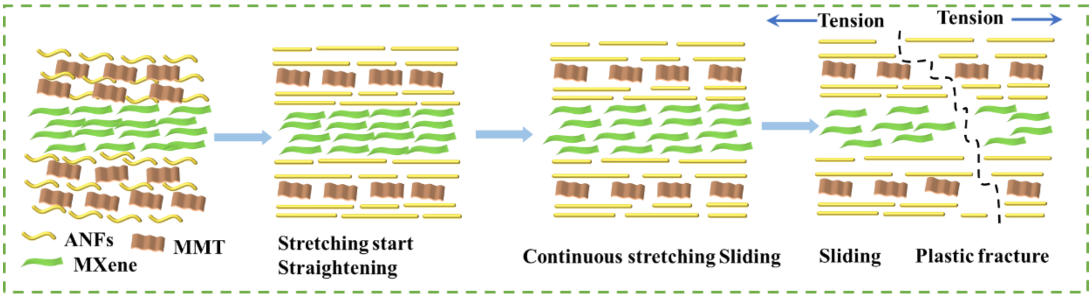

Fig. S15 Proposed fracture process of p-n segmented core-shell TE fiber

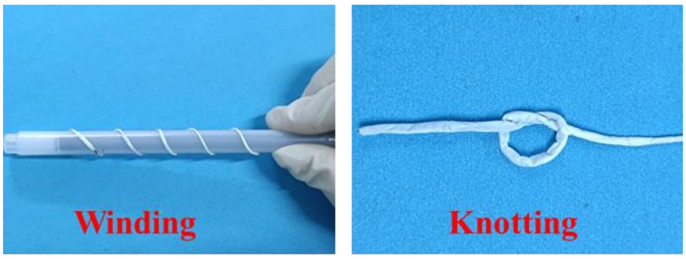

Fig. S16 Super flexibility of the p-n segmented core-shell TE fiber including winding and bending

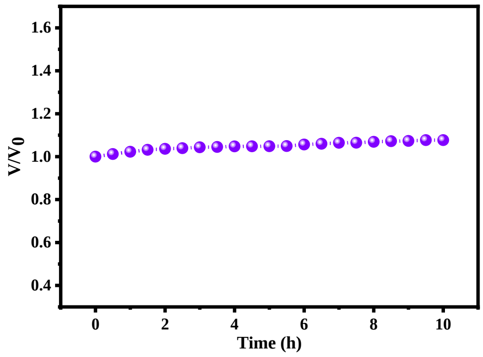

Fig. S17 The effect of washing times on output voltage of TET (0.5 h for each washing cycle)

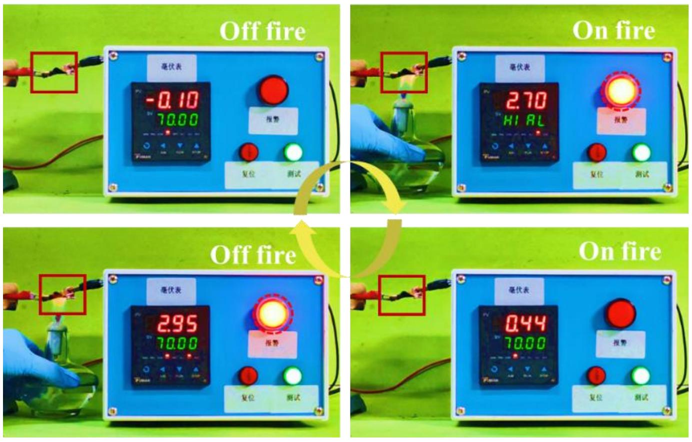

Fig. S18 TET exhibited rapid and repeated fire warning capability when exposed to flame

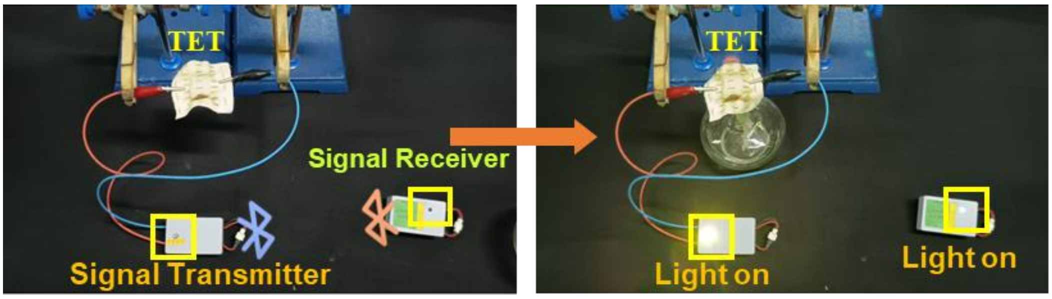

Fig. S19 Self-powered wireless fire warning test for TET indoors

Table S1 The standard for dividing the performance of aerogel fiber

Grade	Tensile strength (MPa)	Conductivity (mS/m)	Diameter (mm)	Knittability	Flexibility	Porosity (%)
1	<1	<1	<0.1			< 90
2	1-2	2-8	0.1-0.4	Unknittable	Inflexible	91-92
3	3-5	8-10	0.5-0.8	Knittable	flexible	93-95
4	6-10	11-15	0.9-1			96-98
5	>10	>15	>1			> 98
